# Development of Soft sEMG Sensing Structures Using 3D-Printing Technologies †

**DOI:** 10.3390/s20154292

**Published:** 2020-07-31

**Authors:** Gerjan Wolterink, Pedro Dias, Remco G. P. Sanders, Frodo Muijzer, Bert-Jan van Beijnum, Peter Veltink, Gijs Krijnen

**Affiliations:** 1 Robotics and Mechatronics Group (RAM), University of Twente, 7500 AE Enschede, The Netherlands; pedrorafael.dias@hotmail.com (P.D.); r.g.p.sanders@utwente.nl (R.G.P.S.); gijs.krijnen@utwente.nl (G.K.); 2 Biomedical Signals and Systems (BSS), University of Twente, 7500 AE Enschede, The Netherlands; b.j.f.vanbeijnum@utwente.nl (B.-J.v.B.); P.H.Veltink@utwente.nl (P.V.); 3 Twente Medical Systems International B.V. (TMSi), 7575 EJ Oldenzaal, The Netherlands; Frodo.Muijzer@tmsi.com

**Keywords:** 3D-printing, thermoplactic polyurethane (TPU), conductive, flexible, soft, EMG, electrodes

## Abstract

3D printing of soft EMG sensing structures enables the creation of personalized sensing structures that can be potentially integrated in prosthetic, assistive and other devices. We developed and characterized flexible carbon-black doped TPU-based sEMG sensing structures. The structures are directly 3D-printed without the need for an additional post-processing step using a low-cost, consumer grade multi-material FDM printer. A comparison between the gold standard Ag/AgCl gel electrodes and the 3D-printed EMG electrodes with a comparable contact area shows that there is no significant difference in the EMG signals’ amplitude. The sensors are capable of distinguishing a variable level of muscle activity of the biceps brachii. Furthermore, as a proof of principle, sEMG data of a 3D-printed 8-electrode band are analyzed using a patten recognition algorithm to recognize hand gestures. This work shows that 3D-printed sEMG electrodes have great potential in practical applications.

## 1. Introduction

Electromyography (EMG) is a technique for measuring the electrical activity produced during muscle contraction. EMG provides useful information about the activity of individual muscles and is therefore widely used in the control of hand and arm assistive devices and prosthesis [1,2,3,4,5,6,7].

Surface electromyography (sEMG) electrodes are currently only available in fixed shapes. Some flexible structures are available, such as the TMSi High Density EMG grids [8]; however, these structures cannot be stretched to adapt to complex anatomy. Therefore, it is often still necessary to place electrodes individually. For everyday patient use of EMG assistive technologies, personalized sensing structures are desirable. Additive manufacturing, best known from 3D printing, enables the creation of personalized low-cost structures, making for a useful tool for prosthetic assistive devices [9]. With the advent of multi-material printers, materials varying in conductivity and mechanical properties can be co-printed, enabling the creation of personalized sensing structures [10,11]. Therefore, 3D-printed sEMG sensing structures will require minimal assembly and are easily scalable. Such structures can be soft and follow body contours, thus not influencing musculoskeletal deformations and greatly improving the comfort of the user and aesthetics of the device.

Although other methods, such as conductive fabrics [12] or screen printing [13], allow for the fabrication of flexible customized sEMG sensors, 3D-printing has the potential to directly print assistive devices and prostheses that incorporate embedded and customized sEMG electrodes that are directly 3D-printed without the need for additional post-processing steps. Furthermore, these structures could also incorporate other types of 3D-printed sensors, such as force or tactile sensors [10,14,15], and include 3D wire routing and shielding.

The goal of this study is to explore the potential use of additive manufactured EMG electrodes made of flexible carbon doped thermoplastic polyurethane by comparing their performance to conventional electrodes. Furthermore, a multi-electrode setup for use in real-time gesture detection is shown as a proof of principle application for 3D-printed electrodes.

### 1.1. EMG

EMG can be recorded either invasively or non-invasively; sEMG gives information about the global muscle activity and is preferable to intramuscular EMG (iEMG) because of its non-invasive nature. The bandwidth of an sEMG signal lies between 0 and about 500 Hz [2,16,17]. The signal values are in the range between a few microvolts to a millivolt depending on the location and the electrodes [2,3]. Peak values lie in the range between 0.1 and 1 mV [16].

In the body, current is facilitated by ion transport. sEMG electrodes convert the ionic current within the tissue into an electronic current. The electrodes can be classified into two main types: dry and wet electrodes [2]. Both wet and dry electrodes have their particular advantages and disadvantages.

The most commonly used electrodes are silver/silver chlorides gel-electrodes (AgCl electrodes). These wet electrodes approximate a perfectly non-polarizable electrode, which is characterized by the free flow of charge between the electrode and the body [2]. AgCl electrodes are widely available and inexpensive [18,19]. Such electrodes are relatively stable when used in biopotential measurements due to the chemical equilibrium reaction between the silver (Ag) electrode and the silverchloride (AgCl) coating, which provides a stable electrochemical interface between ionic medium and electrode [16]. However, AgCl electrodes are less suited for long-term use, since the electrolytic gel will dehydrate over time and this will influence the signal quality [2,19,20]. Another drawback is that some people are sensitive to the electrolyte gel, causing skin irritation and other allergic reactions [20].

Dry electrodes usually have a higher electrode–skin impedance compared to wet electrodes and are more sensitive to movement artifacts [20]. Dry electrodes have the advantage of being reusable and are more suited for long-term measurements [19]. In electrode arrays, dry electrodes are preferred since electrodes are placed close to each other and conductive gel could create conductive pathways, short-circuiting the electrodes [21]. Dry electrodes can be fabricated using various methods and materials. This makes them useful for integrating into prostheses and other devices. The literature shows electrodes made from semiconductor materials, gold-coated 3D prints [20], metal or metal filled polymers [22], conductive fabrics [23], printed circuit boards (PCB) [19], silver inks printed on flexible polyamide films [5] and carbon-filled rubbers [22,24].

Several studies have shown that dry electrodes can give results comparable to wet electrodes after only a few minutes [25,26]. Pylatiuk et al. [25] performed impedance measurements over 3 h on dry and wet electrode. The measurement showed an increase in skin–electrode impedance for the wet electrodes after 30 min, due to evaporation of the electrolyte. After 150 min, the impedance of these electrodes was higher than that of the dry electrodes, resulting in poorer signal qualities. Dry electrodes showed a high skin–electrode impedance directly after application (100 kΩ to 400kΩ). Due to sweat and moisture build-up, the impedance decreased to values ranging from 40kΩ to 80kΩ after 20 min. Over the long term, dry electrodes even have a stabler impedance, since wet electrodes dry out over time [23,25,27].

Due to the relatively high electrode–skin contact impedance, biopotential amplifiers need to have a high input impedance. Webster et al. stated an average contact impedance of 1 c$m^{2}$ skin of around 200 kΩ at 1 Hz to 200 Ω at 1 MHz [16]. Biopotential amplifiers nowadays have input impedances ranging over 100 [28]. An input impedance that is as high as possible is preferred since this also reduces noise caused by impedance mismatch, which generates a differential signal of the common mode voltage. Other noise prevention methods include (active) shielding of the wires between the electrode and the amplifier.

When 3D-printing electrodes, it is preferable to make dry electrodes, since dry electrodes are more suited for long-term measurements and because of hygienic reasons. Since biopotential amplifiers nowadays have large input impedances, 3D-printed electrodes do not need to be made from highly conductive materials.

### 1.2. Conductive 3D Printing

Both 3D-printing technologies and the number of available materials have grown significantly in recent years, while the cost of both continues to decrease. To 3D-print sensing structures, dielectric and conductive materials need to be combined into single structures. Two main methods to embed a conductive material into a 3D-printed structure are filling and direct printing.

The first method adds conductive properties to the material by filling channels or cavities in a printed object with a conductive filler. Filling is usually an extra processing step that takes place after the printing process [29,30,31]. The advantages of this method are the wide variety of conductive fillers and printing techniques that are available. However, most conductive fillers have a high viscosity, resulting in large pressure drops and long filling times, limiting the channel maximum length, minimal cross-section and complexity. Additionally premature solidification of the filler can strongly hamper the filling process. The creation of extensive conductive networks is furthermore limited by the difficulties involved in the removal of the channel support material and complications such as the formation of bubbles [32].

Direct conductive 3D-printing has been demonstrated with the use direct-ink writing using silver particle infused inks or elastomers [33,34,35,36]. Fused deposition modeling (FDM) and stereo lithography (SLA) are commercially available printing technologies that allow direct conductive 3D-printing. SLA is a photo-curing printing method where a liquid resin is solidified layer by layer. Although flexible conductive SLA-printed structures, based on ionic composite hydrogels, have been demonstrated [37], this technology is currently not suitable for the development of multi-electrode structures, since SLA is limited to a single material [14]. FDM is an extrusion-based 3D printing technology in which a thermoplastic material in the form of a filament is pushed through a heated nozzle in which the material is melted. The nozzle deposits a thin layer of molten material on a predefined path in the xy-plane. To create structures extending in the third dimension (*z*-direction) as well, the process is repeated, building up the model layer by layer. To enable the deposition of multiple materials, the printer is fitted with multiple extruders and nozzles.

Models made using FDM printing are usually made from a rigid thermoplastic, such as polylactic acid (PLA), acrylonitrile butadiene styrene (ABS), glycol-modified PET (PETG), polyvinyl alcohol (PVA) or nylon. However, there is a growing variety of flexible materials available. Most of these materials are thermoplastic polyurethanes (TPU); commonly know materials are NinjaFlex and PolyFlex [38,39]. Softer flexible materials are X60 [40] and the Lay-Fomm or Lay-Gell filaments, which become flexible after dissolving the PVA compound, leaving a porous soft structure [41].

To print conductive structures using FDM printing technologies, the raw thermoplastic material is blended with conductive particles such as carbon black. Currently only a few conductive materials are available; see Table 1. PI-ETPU is currently one of two commercially available filaments that have flexible properties (Young’s modulus of 12 MPa) and a low shore hardness of 95 A [42].

An increase of the volume fraction of the conductive particles will reduce the resistivity of the material as modeled by percolation theory. The model predicts that the resistivity of the material (ρ) decreases in response to an increasing filler concentration (*m*) [33,49,50]:

(1) $$\rho =\rho_{0}{\left(m-m_{c}\right)}^{-\beta }\;\;\mathrm{for}\;m>m_{c}$$

where $m_{c}$ is the critical concentration of the conductive filler. The value of the critical exponent (β) depends on the type of particles. Since the decrease of the resistivity is exponential, the effect of the conductive filler concentration will become substantially less as the concentration increases. Due to the decrease in elastic properties in response to increasing filler content [51], a compromise between the resistivity reduction and flexibility needs to be made.

## 2. Methods

### 2.1. Electrode Impedance

To gain insight into the impedance of the EMG electrodes, a pair of 3D-printed electrodes (D10_IED20) and AgCl electrodes were placed on a cloth rinsed in a 0.9% NaCl solution. The measurement was performed in three- and four-point configurations using an LCR meter (HP4284A). The measurement frequency ranged from 20 Hz to 2.5 kHz.

### 2.2. Side-by-Side sEMG Electrode Characterization

**Figure 1:**
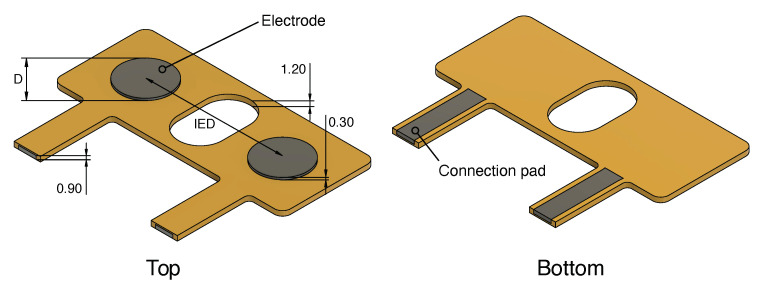
CAD drawing of the electrodes; grey parts are conductive, orange part are dielectric. The dimension are indicated in mm. The inter-electrode distance (IED) and the diameter (D) are variable and listed in Table 2.

To characterize the performance of 3D-printed electrodes compared to commercial electrodes, and to gain insight into the influence of electrode size on the EMG signal, sensing structures with varying geometry were 3D-printed. These sensing structures were designed using Fusion 360 (Autodesk, San Rafael, CA, USA). Figure 1 shows a CAD drawing of the sensing structures. Parts shown in dark gray are made from conductive PI-ETPU, whereas the orange parts are made from non-conductive Ninjaflex and are used as insulation and to keep the electrodes at a fixed distance. Both PI-ETPU and NinjaFlex are flexible materials with a Young’s modulus of 12 MPa and a Shore hardness of 95A for PI-ETPU and 85A for NinjaFlex. The sensing structures of various electrode diameters and inter-electrode distances (IED) are listed in Table 2. The thicknesses (*z*-direction) are equal for all sensing structures and are indicated in Figure 1. The material cost of the sensing structures is estimated to be between €0.10 for the smallest and €0.20 for the largest structure.

**Figure 2:**
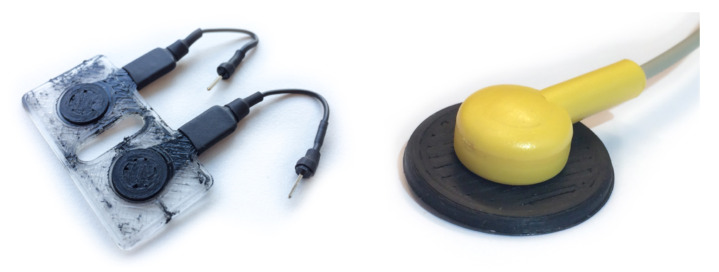
(**Left**): 3D-printed and assembled sensing structure (D10_IED20). (**Right**): Connected 3D-printed snap electrode.

The electrode structures were printed using a modified FlashForge Creator Pro FDM printer (FlashForge Corporation, Jinhua City, Zhejiang Province, China), fitted with two direct drive extruders suited for printing flexible materials (Flexion Extruder, Longmont, CO, USA). A personal computer running slicer software (Simplify3D, Inc., Cincinnati, OH, USA) sliced the CAD model and managed the control of the printer. The structures were printed with a layer height of 150 μm. To compensate for possible errors in the bed leveling, the first layer was printed at a layer height of 300 μm. The nozzle diameter used to extrude NinjaFlex was 600 μm; PI-ETPU was printed using a wider nozzle of 800 μm to prevent blockage. Connections to the sensing structures were made by melting a stranded copper wire onto the connection pads. Shrink tubing was used for insulation and strain relief. The other side of the 20 mm-long unshielded wire interfaces with the shielded wires of the biopotential amplifier. Figure 2 (left) shows an assembled D10_IED20 electrode.

**Figure 3:**
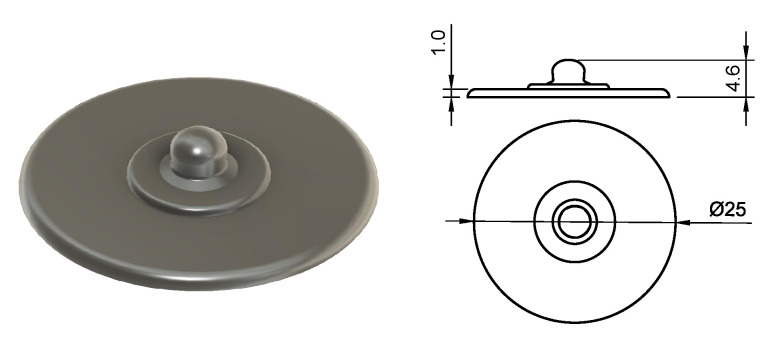
CAD drawing and dimensions of the snap electrode.

For single electrode applications, a snap button electrode was designed; see Figure 2 (right) and Figure 3. This type of connector allows for easy connection since the snap button is a standardized connection used in many commercial EMG systems.

#### 2.2.1. Study Design

Six healthy adult (18+) volunteers participated in the measurements, which were approved by the faculty ethics committee. Before application of the EMG sensing structures, the skin around the biceps brachii is prepared according to SENIAM recommendations [18,52]. If hair is present, the skin is shaved. Next, both the skin and the printed electrodes are cleaned using alcohol. After the alcohol has evaporated, the sensing structures are placed above the biceps brachii according to SENIAM recommendations. The printed snap electrodes function as patient ground and are placed on the wrist of the opposite arm. To provide better electrode-to-skin contact, the electrode is moistened using a cotton swab dipped in tap water. The electrodes are connected to a TMSi Refa (Twente Medical Systems International B.V., Oldenzaal, The Netherlands) biopotential amplifier. This amplifier is connected to a computer via a bidirectional optical fiber. The EMG data are sampled at 1250 Hz, and are visualized and stored using the TMSi Interface for Matlab.

For the first measurement, the subjects were asked to perform three short isometric contractions of 1 s per contraction. To help with the isometric contraction, the subjects were asked to try and lift a table fixed to the floor. The voluntary contraction was measured by asking the subjects to perform elbow flexion followed by extension without any load (concentric contraction); this movement was repeated 3 times. The data obtained from this recording were shown to the researcher in real-time and used to validate the setup and electrode placement.

**Figure 4:**
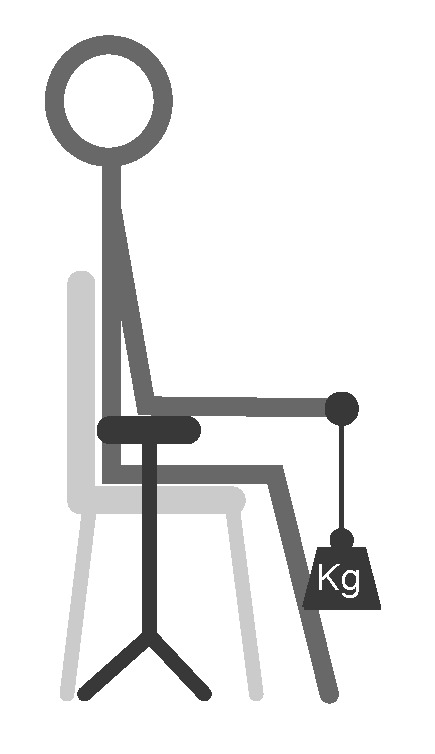
Position of the subject performing the load experiment. The elbow is placed on an armrest, and the load was increased from 0 kg to 5 kg every 10 s.

The second measurement was the load test. The subjects were asked to place their elbow on an armrest and to hold a handle that was attached to a load; see Figure 4. At the start of the measurement, the subjects had to keep this position with no load on the handle. After 10 s the load was increased by 1 kg and the subjects had to keep their arm in position, causing the biceps to contract. Every 10 s, the load was increased by 1 kg until the total load reached 5 kg. Recordings from these tests give information about the signal properties at various loads and help to determine if the printed sensing structures are capable of detecting several levels of muscle activity.

**Figure 5:**
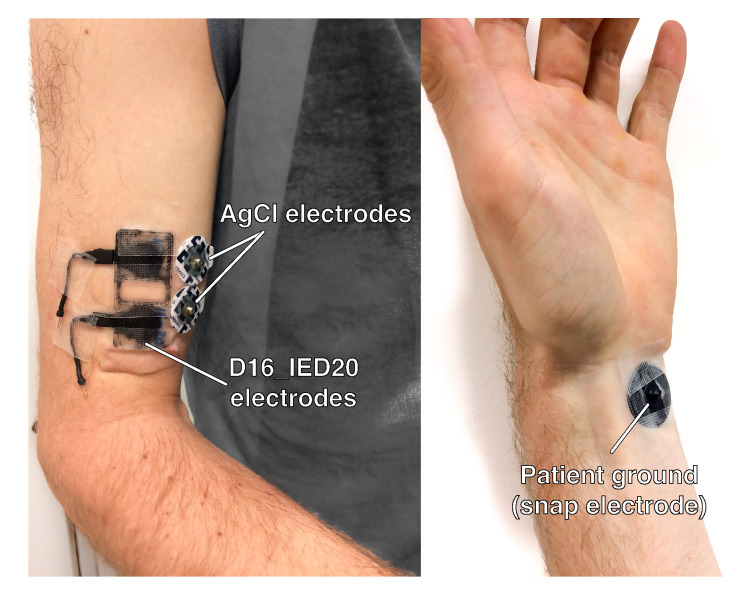
Placement of the electrodes on the subject biceps and wrist.

During both measurements, the subjects remained seated. Both measurements were repeated for different electrode setups (though in all setups the snap electrode was used as the patient ground). The first set of measurements compared the gold standard AgCl electrodes (Covidien, Ireland) and the 3D-printed electrodes. Therefore, the AgCl and printed sensing structures were placed side-by-side on the biceps (Figure 5). The AgCl electrodes were placed next to each other, resulting in an IED of 20 mm. The cross-section of the electrolyte is 16 mm, and therefore the comparison was made using the D16_IED20 printed electrode. The measurements were repeated one by one with various sets of sensing structures as listed in Table 2. The structures were tested in both dry and in wet conditions; for the wet condition the electrode was moisturized using a cotton swab dipped in tap water.

#### 2.2.2. Data Processing

The data were loaded into Matlab and further processed. First, the drift and movement artifacts were removed from the signal by a 20 Hz corner frequency high-pass filter (3rd-order Butterworth). Next, a 50 Hz notch filter was applied to the signal. The higher frequencies were filtered out using a 250 Hz corner frequency low pass-filter (3rd-order Butterworth). Finally, the envelope of the signal was taken using a moving RMS window of 25 samples. The data from the load test were cut into windows of 5 s, each representing a load ranging from 0 kg to 5 kg. The first window starts at 2.5 s, the second at 12.5, and so on. To compare the signals, the mean and the standard error of the RMS values were calculated for each load. To compare between subjects the mean of each load sample was normalized using the mean of the 5 kg load sample of each subject.

#### 2.2.3. Statistical Analysis

From the time domain data, the mean (μ) is given by the sum of all elements ($x_{i}$) of the envelope divided by the number of elements (*n*):

(2) $$\mu =\frac{1}{n}\overset{n}{\underset{i=1}{\sum }}x_{i}$$

To prove, or reject, the difference between AgCl and TPU electrodes, the mean envelope per electrode was taken per subject of the 5 kg sample. Next, the values of the AgCl and the 3D-printed electrode were tested using a paired *t*-test. The increase in muscle activity was proven by taking the mean envelope per subject, per load, of one electrode and testing this against the values of the next load using a paired *t*-test. The same statistical analysis procedure was performed between the various printed electrodes.

### 2.3. Functional Analysis: Classifier Approach

**Figure 6:**
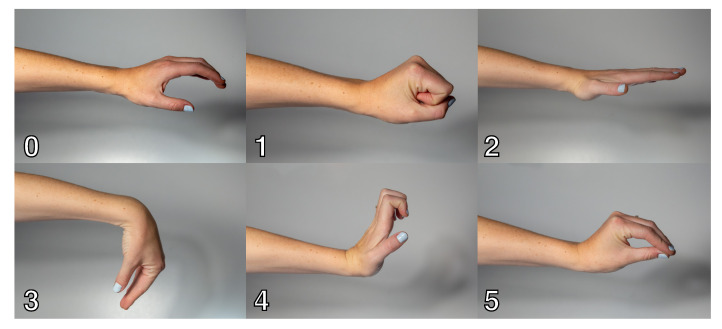
The six classes: rest, fist, hand extension, wrist flexion, wrist extension and pinch grasp.

**Figure 7:**
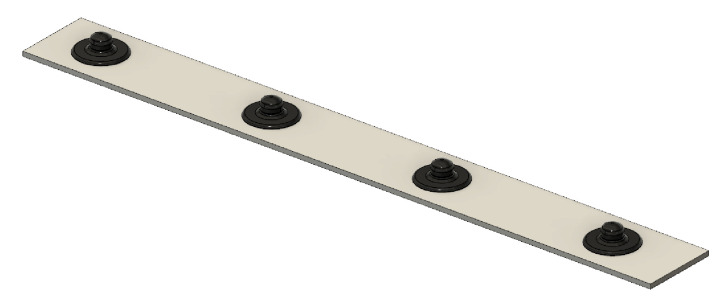
Armband design: the 8-electrode armband consists of two of the illustrated structures.

To demonstrate a real life application, the 3D-printed electrodes, in eight-electrode-structure, were used in a real-time gesture detection test. The electrodes were placed on the lower arm to detect six classes representing six hand positions, as shown in Figure 6: rest, fist, hand extension, wrist flexion, wrist extension and pinch grasp. Due to size limitations of the 3D printer, the 8-electrode armband was made by combining two 4-electrode structures, as illustrated in Figure 7. Each of these structures’ dimensions are 135 × 15 × 1 mm in which the electrodes, with a diameter of 10 mm, are equally spaced.

**Figure 8:**
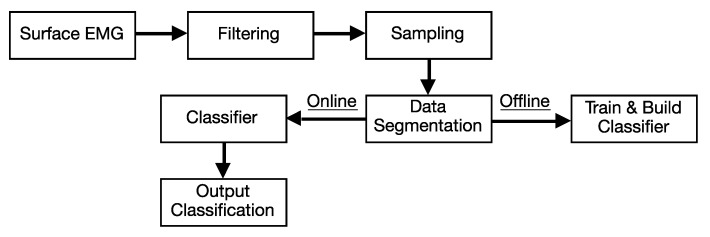
Pipeline from signal acquisition to classification.

Figure 8 presents the pipeline followed from the recording of EMG signals to one of the six classes as shown in Figure 6. The signals are filtered using a discrete-time, infinite impulse response (IIR) fourth-order 5 Hz to 450 Hz bandpass Butterworth filter of direct-form II [53]. Next, the signal is segmented, using 50% overlapping windows of 200 ms. In each window, data segmentation is performed through the computation of the mean absolute value of each of the sensors.

Classification of movements was performed by using linear discriminant analysis (LDA) and support vector machine (SVM) algorithms. The two algorithms were chosen since LDA is fast and has low computational effort, and SVM is more powerful and might allow for better recognition between gestures.

The functional evaluation of the 3D-printed electrode structure is based on the classification accuracy of the 3D-printed eight-electrode structure in comparison to eight AgCl electrodes. Three healthy adult (18+) volunteers participated in the measurement, which was approved by the faculty’s ethics committee. The subjects were placed in a comfortable position, either sitting or standing, but with the notion that all the movement training should be performed with the arm in the same position. The skin was prepared according to SENIAM recommendations [52] and the electrode bands were placed on the right arm below the elbow. Each electrode was connected to the EMG amplifier. The patient ground of the device, consisting of a damp cloth wristband with silver wires, was placed on the left arm. Next, the following protocol is executed:

- Inactivity: for 9 s, the subject stands still to obtain the EMG in the neutral position.
- The following process is repeated for each class:
  - Determining maximum voluntary contraction (MVC): 9 s recording with 3 s of inactivity and 6 s of full contraction.
  - 3 s of inactivity followed by 6 s of contraction at 30%, 50% and 70% of the previously determined MVC. The subject receives real-time feedback about the target EMG and MVC.
- Offline generation of the classifier: building the LDA and SVM classifiers. Performing a 10-fold cross validation using the training data to obtain the confusion matrix.
- Online testing of the classifier: real-time EMG data are analyzed by the classifier to get real-time feedback of the user’s gesture.

This procedure was repeated twice, once for the printed electrodes and once for the AgCl electrodes.

## 3. Results

### 3.1. Impedance Measurements

**Figure 9:**
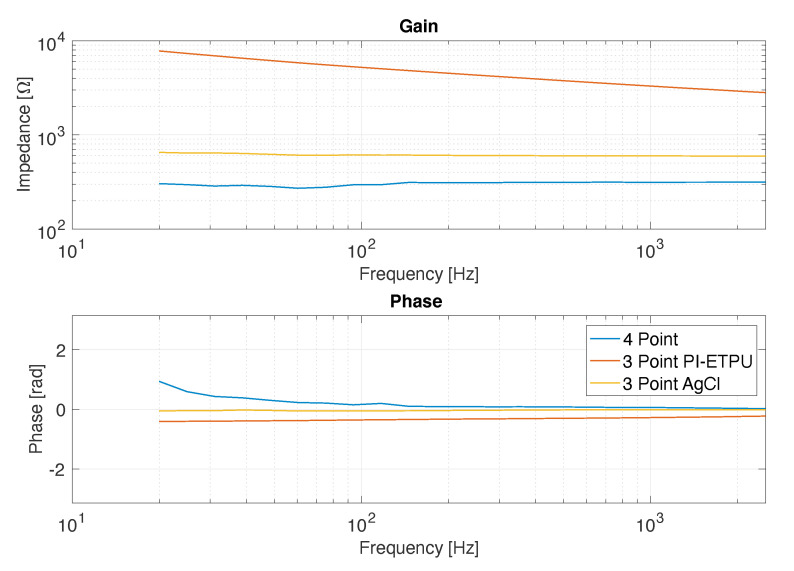
Impedance of the ETPU and AgCl electrodes in contact with 0.9% NaCl solution (3-point) and 0.9% NaCl solution (4-point).

Figure 9 shows the result of the impedance measurements in the three- and four-point configurations. Clearly, the 3-point measurements indicate a higher impedance compared to 4-point measurements, which only showed the impedance of the 0.9% NaCl solution, pointing to high contact impedance.

### 3.2. Side-by-Side sEMG Electrode Characterization

#### 3.2.1. Printed vs. Conventional AgCl Electrodes

**Figure 10:**
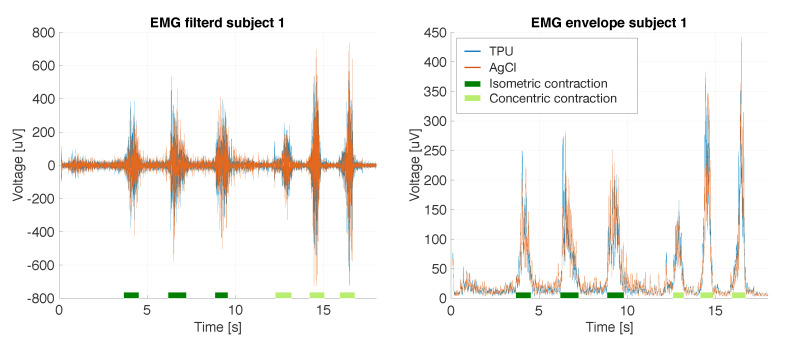
EMG measured by the printed D16_IED20 and AgCl electrodes for a given subject performing three isometric contractions followed by three concentric contractions.

The time-domain data captured using the D16_IED20 and the AgCl electrodes for a given subject, performing three isometric contractions followed by three concentric contractions, are shown in Figure 10. The figures suggest that the signals were highly correlated. The noise in the envelope plot was around 15 μV and the isometric contractions peaked at 250 μV. The concentric contractions peaked at just over 250 μV, 370 μV and 440 μV.

**Figure 11:**
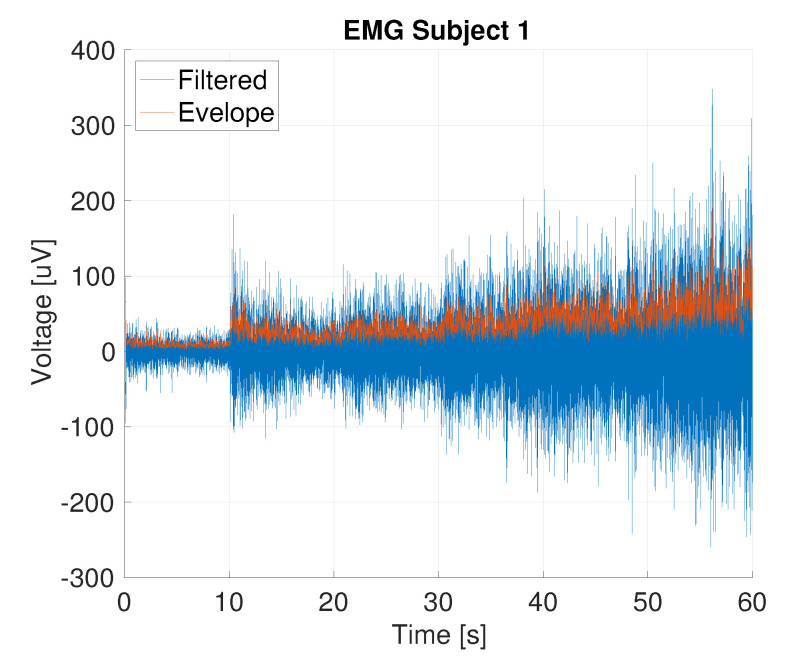
EMG recording and its envelope from the load test of subject 1 (D16_IED20 electrode); the load was increased from 0 kg to 5 kg in ten second intervals.

**Figure 12:**
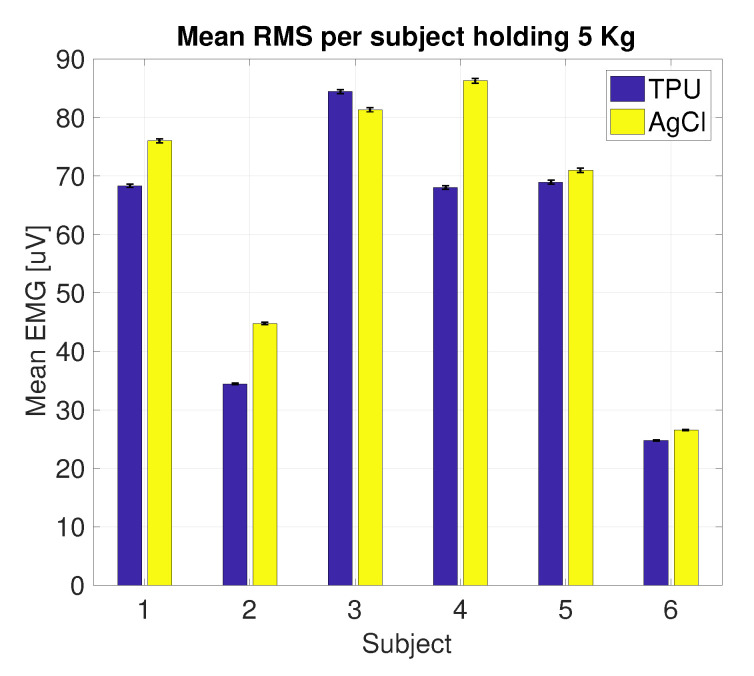
Normalized mean envelope value and standard deviation of all subjects performing the 5 kg load test.

**Figure 13:**
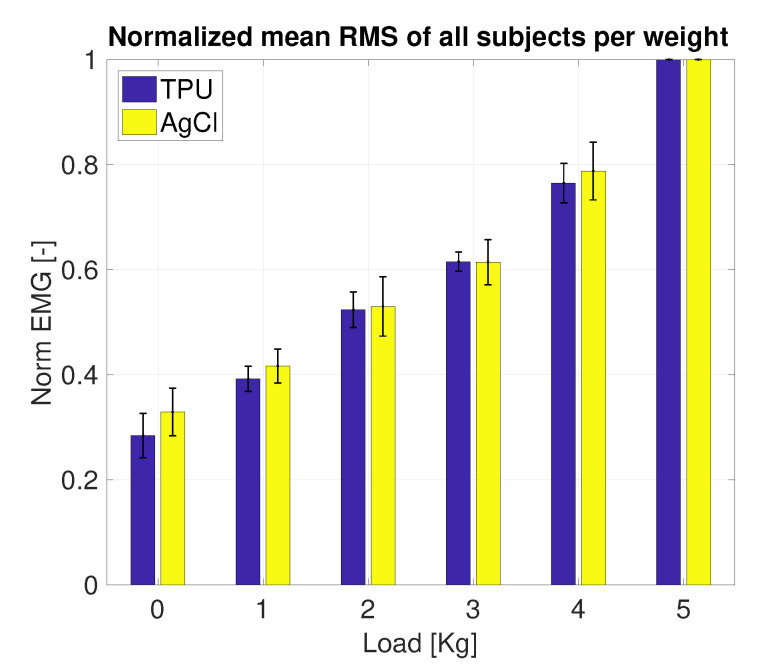
Mean envelope and standard deviation of all subject per load.

Figure 11 shows the filtered signal and the corresponding envelope of one subject performing the load test, measured using the printed electrode. The mean envelope and the corresponding standard deviation per subject per electrode are shown in Figure 12. For all subjects, except subject 3, the mean envelope of the AgCl was significantly (p<0.01) higher; for subject 3 the printed electrodes mean envelope was significantly higher (p<0.01). Figure 13 shows the mean EMG normalized using the 5 kg load sample. The normalized mean envelope value increased significantly between each load sample for both the TPU (p<0.02) and AgCl (p<0.05) electrodes.

#### 3.2.2. Electrode Size

**Figure 14:**
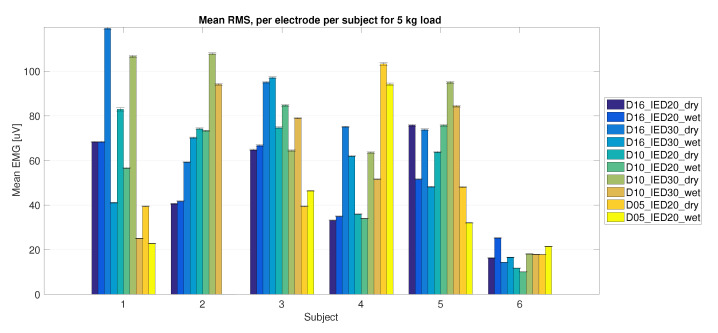
Mean envelope of the 5 kg load sample of each electrode size in wet and dry conditions, shown per subject.

Tests using the smallest electrode structures with an inter-electrode distance of 10 mm (D05_IED10) in both wet and dry conditions only captured EMG from one subject; therefore these data are not shown in the results. A comparison between electrodes per subject lifting a 5 kg load is shown in Figure 14. For subject 2 the EMG signals could not be measured with the smallest electrode structure (D05_IED20) under both dry and wet conditions. Overall the figure shows no clear trend in performance differences between electrode sizes, IED and between dry and wet conditions.

**Figure 15:**
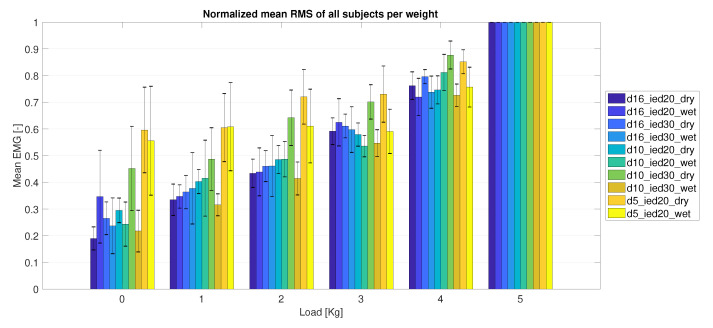
Mean envelope of each electrode obtained for all subjects, shown per load sample.

**Figure 16:**
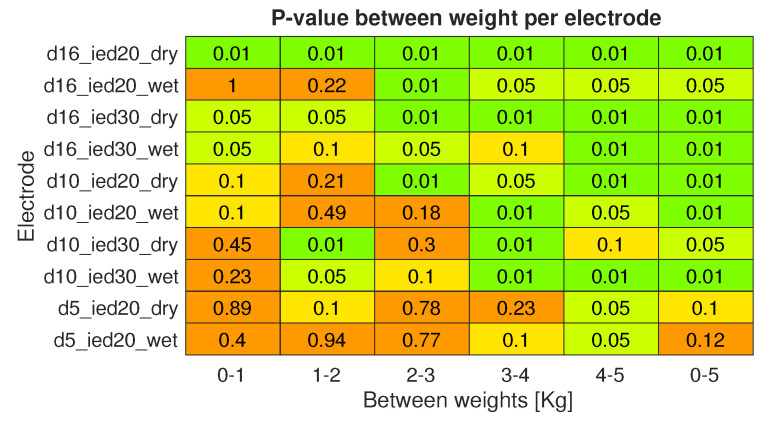
Probability value of all subjects between different load samples for all tested electrodes sizes and conditions (0.01, 0.05 and 0.1 indicate a *p*-value below the subsequent value).

Figure 15 shows the mean envelope, normalized using the 5 kg load sample, over all subjects, per weight. Figure 16 shows the difference per electrode between weights and corresponding *p*-value. These results show that most measurements using the largest electrodes with a diameter of 16 mm (D16_IEDXX_XX) were capable of detecting a significant increase in muscle activity when the load was raised by 1 kg. The medium-size electrodes with a diameter of 10 mm (D10_IEDXX_XX) had less observed significant differences. For the smallest size (D05_IED20_XX), increases and decreases in the mean envelope were most probably coincidental. The difference in mean envelope between 0 kg to 5 kg load data for electrodes of diameters 16 mm and 10 mm was significant. However, for electrodes of a 5 mm diameter, these differences were less significant; see Figure 16 for *p*-values.

### 3.3. Functional Analysis

**Figure 17:**
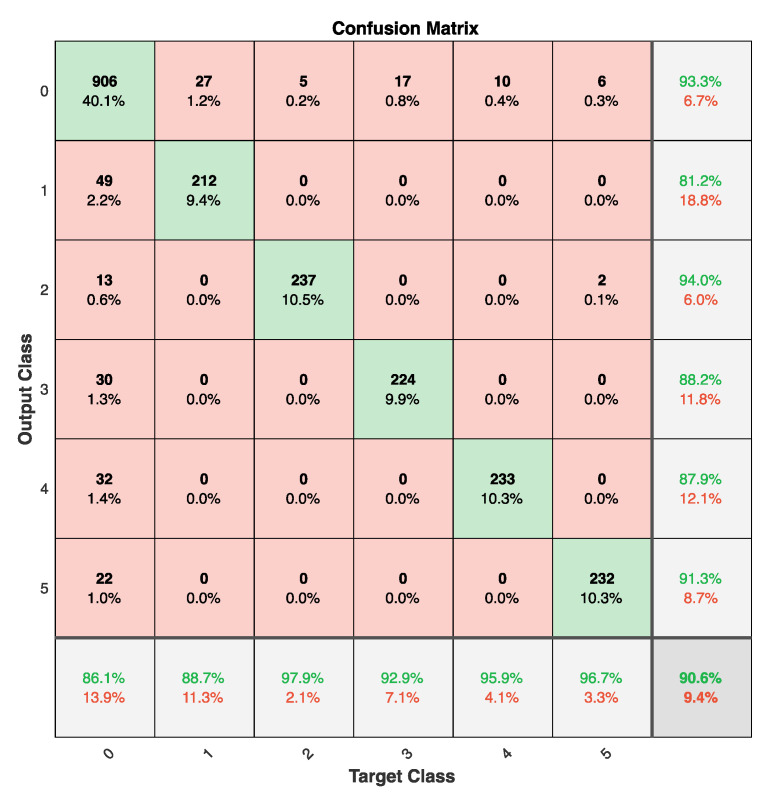
Confusion matrix of subject one SVM classifier for the TPU electrodes.

The functional evaluation of the 3D-printed electrode structures in comparison to the AgCl electrodes is based on the classification accuracy. Table 3 shows the classification accuracy of each subject for each classifier. Confusion matrices show that in most cases, misclassifications were made with respect to the rest state (class 0). These misclassifications where both false positives and false negatives. Figure 17 shows the confusion matrix of the SVM classifier for the printed electrodes on subject 1.

## 4. Discussion

### 4.1. FDM Printing Process

Some spots of the conductive TPU were seen in the dielectric part (Figure 2). These spots were caused by material oozing from the paused nozzle moving over the structure together with the active nozzle. Oozing was kept to a minimum by fine-tuning the slicer setting for retraction and temperature. However, further improvements need to be made by improving the printer’s mechanical design, such as incorporating retractable nozzles or adding the capability to park nozzles that are not in use. The second option gives the largest freedom in expanding the number of nozzles and materials.

Due to the nature of the FDM printing process, structures are built up from fields of parallel lines that are stacked onto each other. The contact interfaces between these lines and layers cause the conductivity of the printed structure to be anisotropic [15,54], manly reducing the conductivity in the *z*-direction [15]. Further study of the anisotropy of the material will lead to further optimized printing patterns and electrode design.

### 4.2. Contact Interface

The four-point measurement (Figure 9) shows that the contribution of the medium is about 20 times lower than the electrode and contact interface resistance that are included in the three-point measurement of the printed electrode. The obtained impedance of the printed electrode in combination with the interface ranged from 7.8 kΩ at 20 Hz to 4.3 kΩ at 250 Hz, and the impedance of the AgCl ranged from 650 Ω to 600 Ω. These values are within the range of carbon-loading silicone rubber electrodes and AgCl electrodes reported by Webster et al. [16]. Due to the high input impedance (over 100 kΩ) of the used biopotential amplifier, the relatively high electrode electrolyte interface of the printed electrode should not influence the signal quality. However, it is likely that the electrode-to-skin interface impedance in dry circumstances is higher than in the situation tested with the electrolyte solution. Furthermore, the skin’s epidermis and subcutaneous layers will increase the electrode-to-skin impedance as well [16].

### 4.3. Electrode Evaluation

The results of the evaluation between the printed and the AgCl electrodes showed that both electrodes are capable of detecting various levels of muscle activity (Figure 13). In Figure 12, five out of six subjects showed a lower amplitude in the mean envelope of the TPU electrodes compared to the AgCl electrodes. However, in this setup it is not possible to validate whether the performance of the AgCl electrodes is better than the printed electrodes due to the fact that the electrodes are placed side-by-side above the biceps brachii; therefore, the electrodes did not measure the exact same volume.

In the electrode size comparison study, the printed electrodes were swapped sequentially after each measurement with a different type varying in diameter and/or inter-electrode distance. Care was taken to place each electrode at the same location as the previous electrode; however, exact repeatable placement was hard to accomplish. Furthermore, there might be a difference between the subjects in subcutaneous fat layers and muscle size, leading to the large individual differences shown in Figure 14. Therefore, this figure does not show a clear relation between electrode size and condition. However, the results strongly suggest that larger electrodes have a performance advantage over smaller electrodes, since the smaller electrodes were less able to detect an amplitude change caused by an increase in load and electrodes with a diameter of 5 mm were unable to obtain a relevant EMG signal in some individuals. Although the performance difference between the wet or dry conditions was not evident in this study, further research on the long-term performance could show a performance increase of the dry electrodes due to moisture build-up [25,26] and a decrease of wet electrode performance due to evaporation [23,25,27].

### 4.4. Hand Posture Classification

The intention of the classifiers is to show the possibility of using 3D-printed electrodes structures to perform online control of prosthetic devices and to compare the 3D-printed electrodes against the AgCl electrodes. Through the results, we were able to prove that the classification accuracy of signal recorded using TPU electrodes is of the same level as the accuracy of the conventional AgCl electrodes. The accuracy came close to values of comparable structures reported by Daley et al. [55] and Li et al. [56]. Moreover, these values can even be improved by looking at the confusion matrices and understand the underlying flaws of the classifier and improving them by going back to the training and rebuilding the classifier. Moreover, the application of more advanced algorithms, such as neural networks, may further improve the classification performance. These algorithms could also facilitate the use of smaller electrodes. The smallest electrodes tested in this study, with a diameter of 5 mm, were capable of measuring EMG signals in some individuals but were unable to resolve the small changes in muscle activity. This information could still be of use in, for example, (high density) grid structures, where the information of multiple sensors could be combined by smart algorithms.

**Figure 18:**
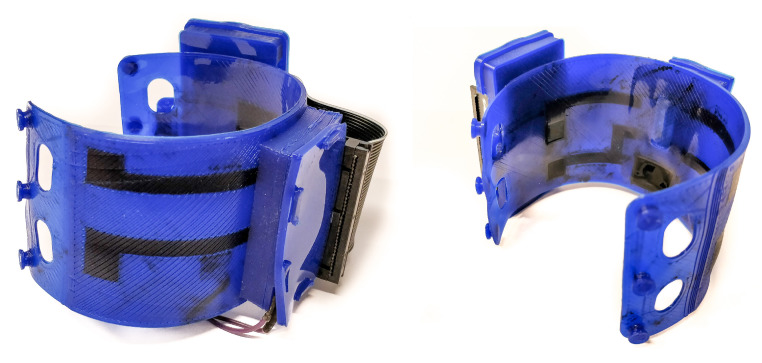
Proof of concept custom-sized 3D-printed EMG armband. The electrodes are internally routed to an EMG amplifier fitted in a housing integrated in the band.

The tested structures presented in this work are relatively simple and flat. However, 3D-printing allows for the fast creation of far more complex structures that could be personalized to the user. A more complex, proof of principle 3D-printed five electrode EMG band is shown in Figure 18; the size and electrode placement of this armband are made specific to one user. Furthermore, the armband houses a small amplifier and microcontroller that could wirelessly transmit the data. These 3D-printed structures require minimal post-production steps and are easily scalable in terms of both the size and number of electrodes, which makes this technique highly accessible. Complex wire routing and shielding can be done in three dimensions inside of the structures, greatly reducing the amount of wiring needed. In the future, sEMG electrodes might be integrated into assistive devices and prostheses that are directly 3D-printed without the need for additional post-processing steps. This allows for easy donning and doffing of the assistive device due to the customized electrode positions and multiple electrodes. Furthermore, the 3D-printed dry electrodes are better-suited for long-time and multiple uses in prostheses since the wet AgCl electrodes can only be used once and will dry out over time.

## 5. Conclusions

This work shows the potential use of additive manufacturing techniques for the creation of flexible personalized sEMG sensing structures. These structures have been successfully printed on a low-cost consumer-grade multi-material FDM printer modified to extrude flexible TPU materials. The material cost of one sensing structure, containing two electrodes, is only €0.20. Comparison between the gold standard AgCl gel electrodes and the printed EMG electrodes with comparable contact areas showed that there is no significant difference in the EMG signal envelope values. This makes 3D-printed electrodes based on conductive TPU highly suitable for practical applications.

The results suggest that 3D-printed electrodes with a size comparable to the gold standard electrodes have a performance advantage compared to smaller 3D-printed electrodes. Large inter-individual differences in the recorded electrode amplitudes were observed both within and between subjects. Even the smallest electrodes, with a diameter of 5 mm, were capable of measuring EMG signals in some individuals. However, these electrodes were shown to be unable to resolve small changes in muscle activity. Overall, this work has shown that 3D-printed electrodes, made from a combination of carbon-doped and regular TPU, display a performance that is largely comparable to the gold-standard AgCl electrodes when paired with the high-quality amplifiers used in this research.

## Figures and Tables

**Table 1:** Overview of commercially available conductive filaments.

Filament/Material	Host	Doping	Volume Resistivity (Ωcm)
Proto-Pasta [43]	PLA	Graphite	30(x,y)115(z)
BlackMagic3D Conductive Graphene [44]	PLA	Graphene	0.6
3D Prima Conductive ABS * [45]	ABS	Carbon fibres	$10^{-7}$–$10^{-6}$
Multi3D, Electrify [46]	Biodegradable polyester	Copper	0.006
PI-ETPU [42]	TPU	Carbon black	<300
EEL [47,48]	TPU	Carbon black	$1.5\times 10^{3}$

* Discontinued product.

**Table 2:** Sensing structures and corresponding dimensions. The electrodes’ names of the bi-polar 3D-printed electrode represent their size, i.e., D16_IED20 has a diameter (D) of  16 mm and inter-electrode distance (IED) of  20 mm.

Name	Diameter (D) (mm)	Inter-Electrode Distance (IED) (mm)
AgCl	16	20
D16_IED20	16	20
D16_IED30	16	30
D10_IED20	10	20
D10_IED30	10	30
D05_IED20	5	20
D05_IED10	5	10
Snap	25	-

**Table 3:** Classification accuracy of each classifier.

	Subject:	1	1	2	3		
Classifier		AgCl	ETPU	ETPU	ETPU	Avg ETPU	Std ETPU
**LDA**	**3 Class**	85.5%	88.6%	78.1%	82.5%	83.1%	5.3%
	**5 Class**	81.6%	86.5%	77.5%	80.2%	81.4%	4.6%
**SVM**	**3 Class**	90.4%	90.9%	81.1%	85.9%	86.0%	4.9%
	**5 Class**	86.8%	90.6%	83.1%	87.1%	86.9%	3.8%
